# Identification of Transcription Factors for Lineage-Specific ESC Differentiation

**DOI:** 10.1016/j.stemcr.2013.10.006

**Published:** 2013-11-27

**Authors:** Kohei Yamamizu, Yulan Piao, Alexei A. Sharov, Veronika Zsiros, Hong Yu, Kazu Nakazawa, David Schlessinger, Minoru S.H. Ko

**Affiliations:** 1 Laboratory of Genetics, National Institute on Aging, National Institutes of Health, Baltimore, MD 21224, USA; 2 Unit on Genetics of Cognition and Behavior, National Institute of Mental Health, National Institutes of Health, Bethesda, MD 20892, USA; 3 Department of Systems Medicine, Sakaguchi Laboratory, Keio University School of Medicine, Tokyo 160-8582, Japan; 4 Japan Science and Technology Agency, CREST, Tokyo160-8582, Japan

## Abstract

A network of transcription factors (TFs) determines cell identity, but identity can be altered by overexpressing a combination of TFs. However, choosing and verifying combinations of TFs for specific cell differentiation have been daunting due to the large number of possible combinations of ∼2,000 TFs. Here, we report the identification of individual TFs for lineage-specific cell differentiation based on the correlation matrix of global gene expression profiles. The overexpression of identified TFs—*Myod1*, *Mef2c*, *Esx1*, *Foxa1*, *Hnf4a*, *Gata2*, *Gata3*, *Myc*, *Elf5*, *Irf2*, *Elf1*, *Sfpi1*, *Ets1*, *Smad7*, *Nr2f1*, *Sox11*, *Dmrt1*, *Sox9*, *Foxg1*, *Sox2*, or *Ascl1*—can direct efficient, specific, and rapid differentiation into myocytes, hepatocytes, blood cells, and neurons. Furthermore, transfection of synthetic mRNAs of TFs generates their appropriate target cells. These results demonstrate both the utility of this approach to identify potent TFs for cell differentiation, and the unanticipated capacity of single TFs directly guides differentiation to specific lineage fates.

## Highlights

• Lineage-determining single TFs are identified based on the correlation matrix
• A proof of concept is demonstrated for ESC differentiation by 21 TFs
• TFs orchestrate global gene expression changes via direct binding to target genes
• Transfections of synthetic TF mRNAs generate desired differentiated cells

## Teaser

Here, Ko and colleagues report that the correlation matrix of global gene expression profiles can identify individual TFs whose overexpression directs efficient, specific, and rapid differentiation into myocytes, hepatocytes, blood cells, and neurons. These results demonstrate that the unanticipated capacity of single TFs directly guides differentiation to specific lineage fates.

## Introduction

One of the goals of regenerative medicine is to generate the desired types of differentiated cells from pluripotent stem cells, such as embryonic stem cells (ESCs) and induced pluripotent stem cells (iPSCs) (Evans and Kaufman, 1981; Martin, 1981; Thomson et al., 1998; Takahashi and Yamanaka, 2006). Common strategies have been the use of a vast knowledge of developmental biology, which provides the information on the sequential requirement of transcription factors (TFs), growth factors, signaling cascades, and cell-to-cell interactions, to optimize the culture conditions for ESC differentiation (Murry and Keller, 2008; Snykers et al., 2009). Particularly, because a network of TFs defines the identity of cells, which can be altered by the forced induction of combination of TFs (Davis et al., 1987; Takahashi and Yamanaka, 2006; Feng et al., 2008; Szabo et al., 2010; Vierbuchen et al., 2010; Caiazzo et al., 2011; Son et al., 2011; Sekiya and Suzuki, 2011; Huang et al., 2011), understanding the structure and dynamics of TF networks may be a sensible first step toward achieving the effective cell differentiation. In developmental biology, it has been well established that TFs generally work in a manner like a cascade: early-acting TFs initiate the differentiation, mid-acting TFs specify the cell lineage, and late-acting TFs complete the process to finally form the maturely differentiated cells (Murry and Keller, 2008; Snykers et al., 2009; Zaret and Grompe, 2008). However, the vast and manifold complexity of TF regulatory mechanisms poses a great challenge in finding a right combination of TFs.

To facilitate the TF network analysis, we and others have applied a systems biology approach (Chuang et al., 2010) to the loss-of-function, i.e., knockout or repress TFs in mouse ESCs (Skarnes et al., 2004; Ivanova et al., 2006; Collins et al., 2007; Nishiyama et al., 2013), followed by the phenotype or global transcriptome analyses. However, the gain-of-function, i.e., overexpression of TFs, approach is more desirable because the alteration of cell identity has thus far been achieved by the forced induction of combination of TFs (Davis et al., 1987; Takahashi and Yamanaka, 2006; Feng et al., 2008; Szabo et al., 2010; Vierbuchen et al., 2010; Caiazzo et al., 2011; Son et al., 2011; Sekiya and Suzuki, 2011; Huang et al., 2011). Therefore, we have established the NIA Mouse ES Cell Bank (Nishiyama et al., 2009; Correa-Cerro et al., 2011), in which each of 137 TFs, i.e., 7%–10% of all TFs encoded in the mouse genome (1,500–2,000 TFs) (Kanamori et al., 2004), can be induced in a tetracycline-regulatable manner. We have measured the global gene expression profiles (i.e., transcriptome) of these ESC lines 48 hr after overexpressing each TF (Correa-Cerro et al., 2011) and generated the correlation matrix by comparing TF-induced gene expression profiles and the expression profiles of a variety of cell types with the Novartis Research Foundation (GNF) (Wu et al., 2009; Su et al., 2002). Of 137 TFs, 63 TFs show a general trend in which overexpression of specific TFs initiated transcriptome shifts toward certain types of differentiated cells, suggesting that this method could predict “master and ancillary regulators” to determine cell fate (see Figure S1 available online).

Here, we propose and test the use of the correlation matrix to predict, validate, and identify potent TFs for cell differentiation. We show a proof of concept by demonstrating direct cell differentiation into target organ cells such as myocytes, hepatocytes, blood cells, and neurons by overexpression of identified single TFs.

## Results

### Prediction of Master and Ancillary TFs for ESC Differentiation

**Figure 1:**
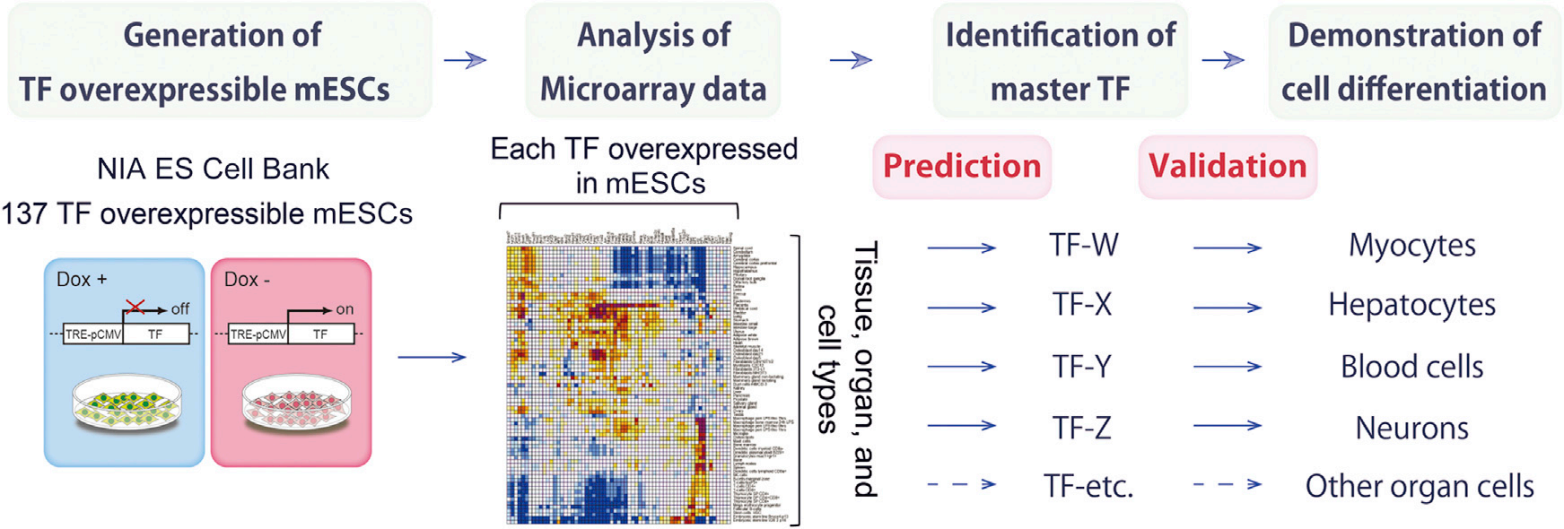
A Schematic Representation of the ESC Differentiation Strategy Based on the Correlation Matrix of Gene Expression Profiles Previously, we have generated the global gene expression profiles obtained by overexpressing single TFs using the NIA Mouse ES Cell Bank, which consists of 137 mouse ESC lines. We have then generated the correlation matrix comparing TF-induced gene expression profiles and the expression profiles of a variety of cell types. TFs predicted by the correlation matrix for specific cell differentiation are selected and subjected for the detailed differentiation assays. See also Figure S1.

Previously, we reported gene expression profiling data that have been generated 48 hr after overexpressing 137 TFs individually in mouse ESCs (Figures 1 and S1) (Nishiyama et al., 2009; Correa-Cerro et al., 2011). Although ESCs remained undifferentiated morphologically, we considered that if a gene could act as a “master regulator” or “ancillary regulator” along a differentiation trajectory (Aiba et al., 2009), it should show corresponding similarities of global gene expression profiles to those in the eventual differentiated cells and tissues. Indeed, we reported that of 137 TFs, 63 TFs show a general trend in the correlation matrix, in which overexpression of specific TFs initiated transcriptome shifts toward certain types of differentiated cells, often negatively correlated with inhibition of different lineages (Figure S1) (Correa-Cerro et al., 2011). This prompted us to consider whether the correlation matrix can be used to identify TFs whose overexpression can differentiate the ESCs into the desired cell types (Figure 1). To test this strategy, here, we chose four types of cell differentiation systems from the correlation matrix and carried out the detailed studies as described below.

### Direct Differentiation into Myocytes

**Figure 2:**
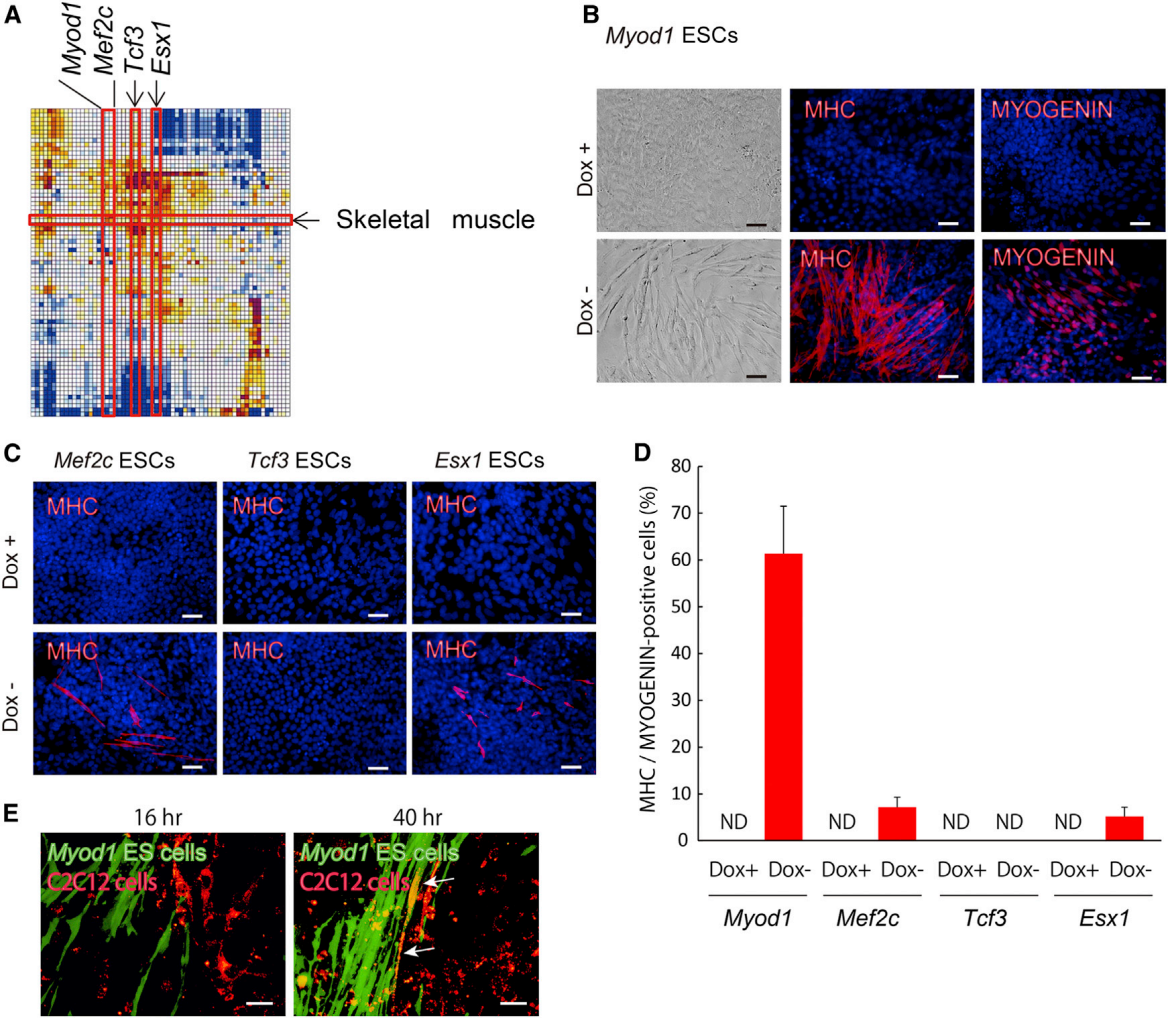
A Single TF Induces Myocytes from ESCs (A) *Myod1*, *Mef2c*, *Tcf3*, and *Esx1* are identified, whose expression shifted the transcriptome of ESCs toward skeletal muscle within the correlation matrix. (B and C) Immunostaining for MHC or MYOGENIN at differentiation for 5 days used ESCs carrying *Myod1*, *Mef2c*, *Tcf3*, or *Esx1* gene. Upper panels show Dox+. Lower panels present Dox−. Scale bars, 200 μm. (D) Quantitative evaluation of MHC^+^/MYOGENIN^+^ cell percentages on day 7 of differentiation used ESCs carrying *Myod1*, *Mef2c*, *Tcf3*, or *Esx1* gene (three independent experiments, SEM). ND, not detected. (E) Analysis of cell fusion between myocytes derived from ESCs and C2C12 cells is shown. White arrows indicate the fusion of myocytes and C2C12 cells. Scale bars, 100 μm. See also Figures S1 and S2.

The correlation matrix showed that *Myod1* was a top-ranked TF, followed by *Mef2c*, *Tcf3*, and *Esx1*, whose overexpression shifted a transcriptome of ESCs toward skeletal muscles (Figures 2A and S1) (Nishiyama et al., 2009; Correa-Cerro et al., 2011). To test this notion, we cultured *Myod1-*, *Mef2c-*, *Tcf3-*, or *Esx1*-inducible ESCs in the standard differentiation medium (DM) in the absence of LIF. The overexpression of *Myod1* dramatically increased the number of cells stained with antibodies against myosin heavy chain (MHC) and MYOGENIN by day 5, confirming the rapid differentiation of ESCs into myocytes (Figures 2B and 2D). The results seem to be consistent with the previous reports that the overexpression of *Myod1* directly converts fibroblast cells, ESCs, or iPSCs into muscle cells (Davis et al., 1987; Dekel et al., 1992; Goudenege et al., 2012; Tanaka et al., 2013). As expected, the overexpression of *Myod1* significantly increased the number of PDGFRα^+^ mesoderm cells, whereas it did not increase FOXA2^+^ endoderm cells and PSA-NCAM^+^ neural progenitors by day 5 (Figure S2). Furthermore, the overexpression of *Mef2c* or *Esx1*, but not *Tcf3*, also generated MHC^+^ and MYOGENIN^+^ myocytes from ESCs (Figures 2C and 2D). The ESC-derived myocytes with *Myod1*-IRES-*Venus* (GFP-variant) overexpression gained the ability to form cell fusion with C2C12 mouse myoblast cells prestained with a red PKH26 dye 40 hr after coculture, indicating some functional maturity of the differentiated cells (Figure 2E).

### Direct Differentiation into Hepatocytes

**Figure 3:**
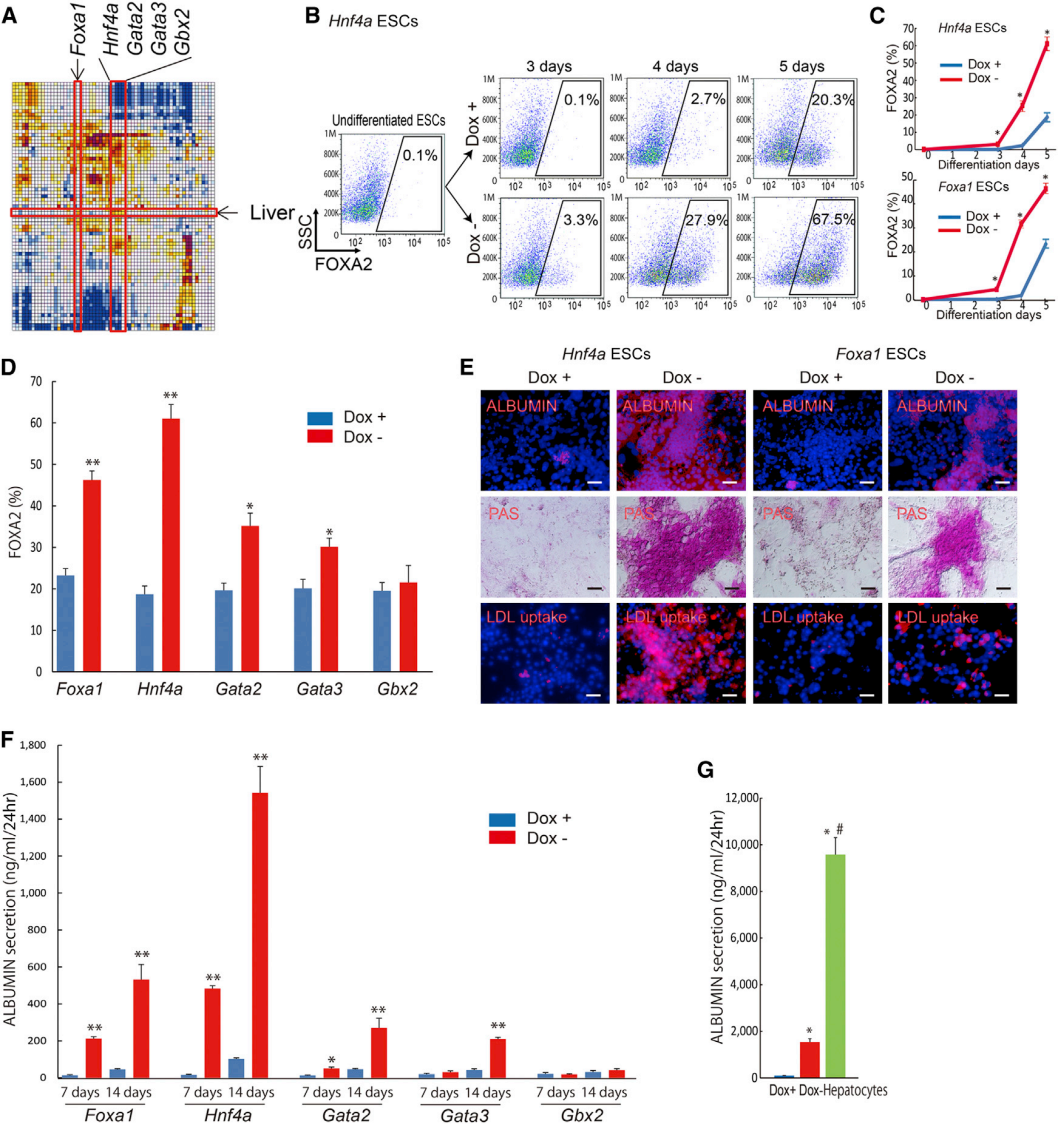
A Single TF Induces Hepatocytes from ESCs (A) *Foxa1*, *Hnf4a*, *Gata2*, *Gata3*, and *Gbx2* are identified for ESC differentiation toward liver. (B and C) FACS analysis for FOXA2 (endoderm marker)^+^ cell appearance used ESCs carrying *Hnf4a*, *Foxa1*, *Gata2*, *Gata3*, or *Gbx2* gene (three independent experiments, SEM; ^∗^p < 0.01 versus Dox+). (D) Quantitative evaluation of FOXA2^+^ cell percentages on day 5 of differentiation used ESCs carrying *Foxa1*, *Hnf4a*, *Gata2*, *Gata3*, or *Gbx2* gene (three independent experiments, SEM; ^∗^p < 0.05 and ^∗∗^p < 0.01 versus Dox+). (E) *Hnf4a-* and *Foxa1-*overexpressing ESCs were assayed for immunostaining of ALBUMIN, PAS staining, and LDL uptake on day 7 of differentiation. Scale bars, 200 μm. (F and G) Amounts of ALBUMIN in the culture media, measured on day 7 and 14 of *Foxa1-*, *Hnf4a-*, *Gata2-*, *Gata3-*, or *Gbx2-*overexpressing ESC differentiation, and primary hepatocytes (three independent experiments, SEM; ^∗^p < 0.05 and ^∗∗^p < 0.01 versus Dox+, #p < 0.01 versus Dox−), are shown. See also Figures S1, S2, and S3.

The correlation matrix showed that *Foxa1* and *Hnf4a* were two top-ranked TFs, followed by *Gata2*, *Gata3*, and *Gbx2*, whose overexpression shifted a transcriptome of ESCs toward liver (Figures 3A and S1) (Nishiyama et al., 2009; Correa-Cerro et al., 2011). Indeed, the overexpression of *Hnf4a* or *Foxa1* rapidly and dramatically increased the proportion of the endoderm cells: FOXA2^+^ cells measured by the FACS; and SOX17- and α-fetoprotein (AFP)^+^ cells measured by the immunofluorescence analyses (Figures 3B–3D and S3A). By contrast, *Hnf4a* did not increase PDGFRα^+^ mesoderm cells and PSA-NCAM^+^ neural progenitors (Figure S2B). As early as day 7, the albumin production was detected in *Hnf4a*- or *Foxa1*-overexpressing cells, but not in the Dox+ control cells (Figure S3A). Use of hepatocyte-specific medium containing hepatocyte growth factor (HGF) and oncostatin M (OSM) from day 3 further increased the fraction of ALBUMIN-producing cells in *Hnf4a*- or *Foxa1*-overexpressing cells, as demonstrated by the Periodic acid-Schiff (PAS) staining, the uptake of low-density lipoproteins (LDLs), and the secretion of ALBUMIN in culture medium on day 7 (Figures 3E and 3F). The overexpression of *Gata2* or *Gata3*, but not *Gbx2*, increased FOXA2^+^ endoderm cells by 5 days after differentiation and generated hepatocytes as demonstrated by the ALBUMIN secretion and PAS staining by 7 and 14 days after differentiation (Figures 3D, 3F, and S3B). By day 14, the amount of secreted ALBUMINs in *Hnf4a-*overexpressing cells reached about one-sixth of that in the primary culture of hepatocytes, suggesting the maturity of the produced hepatocytes (Figure 3G). Interestingly, *Hnf4a* showed more potent effects on hepatocyte differentiation than *Foxa1*, which is consistent with the known timings of these TF actions: *Foxa1* is required in early stage for hepatocyte differentiation, whereas *Hnf4a* is required in late stage during development (Snykers et al., 2009; Zaret and Grompe, 2008; Duncan, 2003). Thus, *Hnf4a* seems to bypass the natural order of TF activation cascades for hepatocyte differentiation and induces hepatocytes directly from ESCs.

### Direct Differentiation into Blood Cells

**Figure 4:**
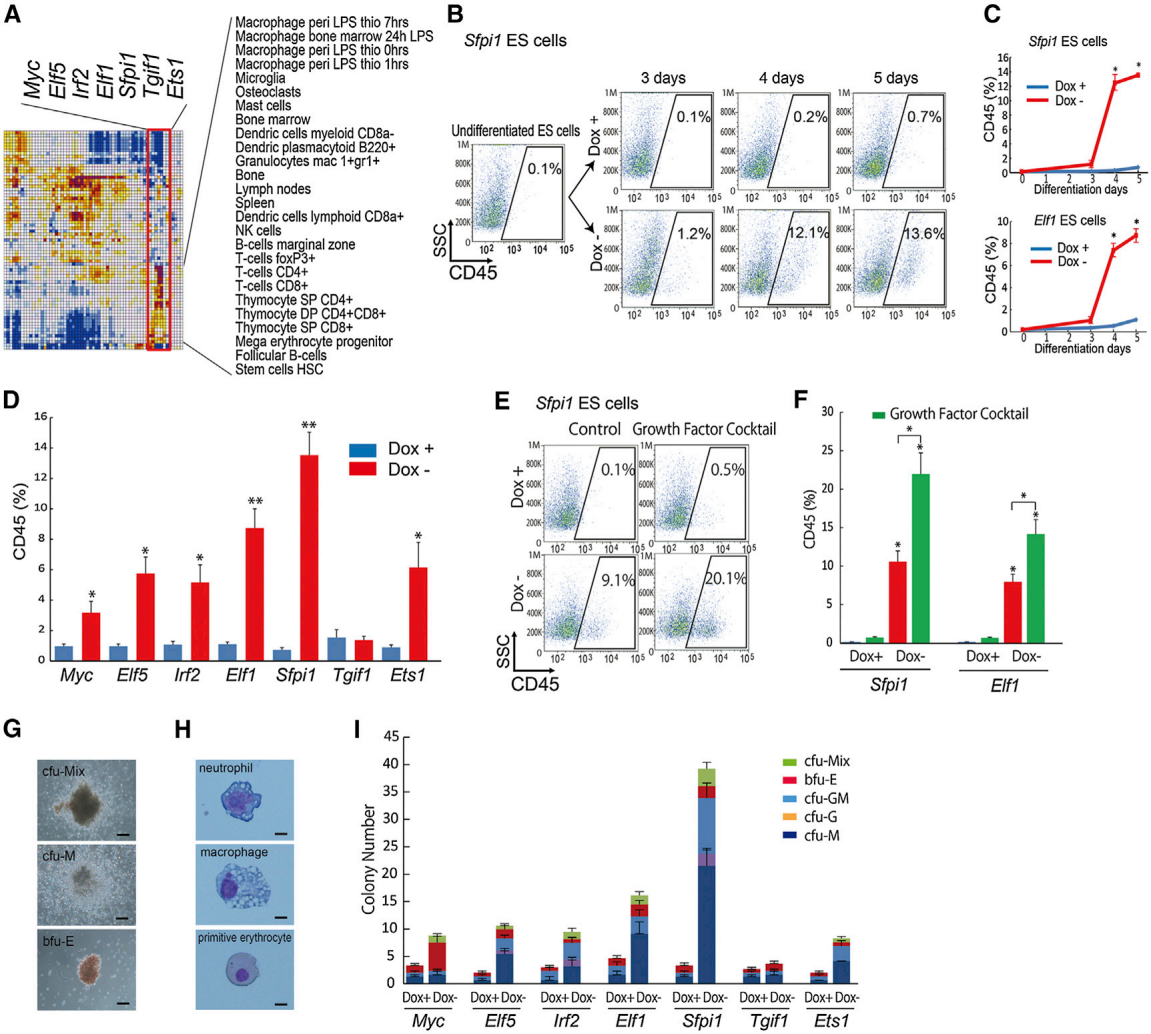
A Single TF Induces Blood Cells from ESCs (A) *Myc*, *Elf5*, *Irf2*, *Elf1*, *Sfpi1*, *Tgif1*, and *Ets1* are identified for ESC differentiation toward blood cells. (B and C) FACS analysis for CD45 (pan-hematopoietic marker)^+^ cell appearance used ESCs carrying *Sfpi1* or *Elf1* gene (three independent experiments, SEM; ^∗^p < 0.01 versus Dox+). (D) Quantitative evaluation of CD45^+^ cell percentages on day 5 of differentiation used ESCs carrying *Myc*, *Elf5*, *Irf2*, *Elf1*, *Sfpi1*, *Tgif1*, or *Ets1* gene by FACS analysis (three independent experiments, SEM; ^∗^p < 0.05 and ^∗∗^p < 0.01 versus Dox+). (E and F) FACS analysis for CD45^+^ cell appearance used ESCs carrying *Sfpi1* or *Elf1* gene with growth factor cocktail (three independent experiments, SEM; ^∗^p < 0.01 versus Dox+). (G) Representative photomicrographs of mix lineage (top panel), macrophage (middle panel), and erythrocyte (bottom panel) at differentiation 11 days after colony-forming assay using ESCs carrying *Sfpi1* gene are presented. Scale bars, 200 μm. cfu-Mix, colony forming unit (cfu)-mix lineage (erythrocyte/granulocyte/macrophage); cfu-M, cfu-macrophage; bfu-E, burst forming unit (bfu)-erythrocyte. (H) May-Giemsa staining used ESCs carrying *Sfpi1* gene. Scale bars, 10 μm. (I) HPC colony numbers in the Dox+ (1 μg/ml) or Dox− condition after 11 days of colony-forming assay are shown (three independent experiments, the total number of colonies, Myc Dox+ was 3.34 ± 1.2 versus Dox− of 8.83 ± 0.88; Elf5 Dox+ was 2.03 ± 0.58 versus Dox− of 10.64 ± 1.53; Irf2 Dox+ was 3.02 ± 0.77 versus Dox− of 9.48 ± 2.31; Elf1 Dox+ was 4.67 ± 1.33 versus Dox− of 16.15 ± 2.52; Sfpi1 Dox+ was 3.34 ± 1.2 versus Dox− of 39.21 ± 3.18; Tgif1 Dox+ was 2.69 ± 0.66 versus Dox− of 3.68 ± 1.51; and Ets1 Dox+ was 2.03 ± 0.58 versus Dox− of 8.28 ± 2.01). cfu-Mix, colony-forming unit (cfu)-mix lineage (erythrocyte/granulocyte/macrophage); bfu-E, burst-forming unit (bfu)-erythrocyte; cfu-GM, cfu-granulocyte/macrophage; cfu-G, cfu granulocyte; cfu-M, cfu macrophage. See also Figure S1.

The correlation matrix showed that *Elf1* and *Sfpi1* (also known as *PU.1*) were two top-ranked TFs, followed by *Myc*, *Elf5*, *Irf2*, *Tgif1*, and *Ets1*, whose overexpression shifted a transcriptome of ESCs toward multiple-lineage blood cells (Figures 4A and S1) (Nishiyama et al., 2009; Correa-Cerro et al., 2011). *Sfpi1* has been known as a TF that plays a critical role in the relatively late phase of hematopoietic lineage specification: specific differentiation of macrophages, granulocytes, and B lymphocytes (Fisher and Scott, 1998). Lesser-known *Elf1* is associated with the development of T cells, especially NKT cells (Choi et al., 2011). Therefore, it is interesting to know whether the correlation matrix-based prediction indeed identifies candidate TFs for hematopoietic differentiation. Upon the overexpression of either *Sfpi1* or *Elf1*, ESCs rapidly and efficiently differentiated into CD45^+^ hematopoietic cells from day 4 of induction with DM (Figures 4B–4D). As expected, the fraction of CD45^+^ cells significantly increased by switching the culture media on day 3 to those containing hematopoietic growth cocktails including the stem cell factor, IL-3, IL-6, IL-11, G-CSF, M-CSF, Flt3 ligand, ERYTHROPOIETIN, and THROMBOPOIETIN (Figures 4E and 4F). The overexpression of *Myc*, *Elf5*, *Irf2*, or *Ets1*, but not *Tgif1*, also produced CD45^+^ hematopoietic cells by day 5 of differentiation (Figure 4D). Furthermore, when the cells with *Myc*, *Elf5*, *Irf2*, *Elf1*, *Sfpi1*, or *Ets1* overexpression were subjected to the colony-forming assay from day 3, these cells were differentiated into macrophages, granulocytes, and primitive erythrocytes after 11 days (Figures 3G–3I), indicating that rationally defined TFs have a potential to produce the multilineage blood cells from ESCs.

### Direct Differentiation into Neurons and Specification of Neural Types

**Figure 5:**
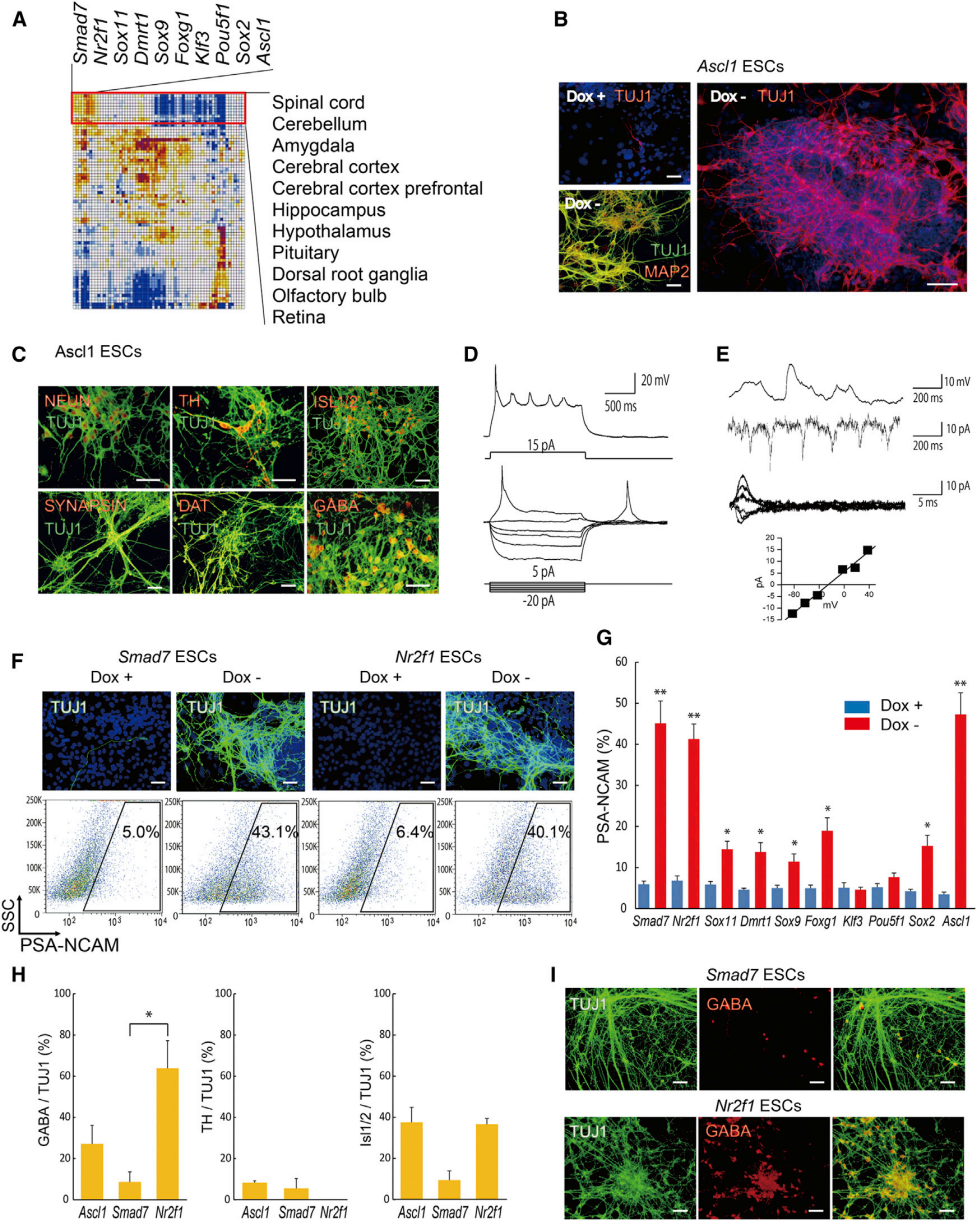
A Single TF Induces Neurons from ESCs and Specification of Neural Types (A) *Smad7*, *Nr2f1*, *Sox11*, *Dmrt1*, *Sox9*, *Foxg1*, *Klf3*, *Pou5f1*, *Sox2*, and *Ascl1* are identified for ESC differentiation toward neural tissues/organs. (B and C) Immunostaining of *Ascl1*-overexpressing ESCs cultured in the DMs for TUJ1, MAP2, NEUN, SYNAPSIN, TH, DAT, ISL1/ISL2, or GABA on day 7 of differentiation is presented. Scale bars, 200 μm. (D) Representative traces show action potentials recorded from neurons induced by *Ascl1* on day 11 of differentiation. (E) Representative traces show postsynaptic currents recorded at −90 mV holding potential. Reversal potential that was calculated from traces was −25.5 mV. (F) Immunostaining of *Smad7*- or *Nr2f1*-overexpressing ESCs for TUJ1 on day 7 of differentiation (top panels) is shown. Scale bars, 200 μm. FACS analysis on day 6 of differentiation for PSA-NCAM (neural progenitor marker)^+^ cell appearance used ESCs carrying *Smad7* or *Nr2f1* gene (bottom panels). (G) Quantitative evaluation of PSA-NCAM^+^ cell percentages used ESCs carrying *Smad7*, *Nr2f1*, *Sox11*, *Dmrt1*, *Sox9*, *Foxg1*, *Klf3*, *Pou5f1*, *Sox2*, or *Ascl1* gene by FACS analysis (three independent experiments, SEM; ^∗^p<0.05 and ^∗∗^p<0.01 versus Dox+). (H) Quantitative evaluation of GABA^+^/TUJ1^+^, TH^+^/TUJ1^+^, and ISL1/ISL2^+^/TUJ1^+^ cell percentages used ESCs carrying *Ascl1*, *Smad7*, and *Nr2f1* by immunostaining (three independent experiments, SEM; ^∗^p < 0.05). (I) Immunostaining of *Smad7*- or *Nr2f1-*overexpressing ESCs for TUJ1 (left panels) and GABA (middle panels) on day 7 of differentiation is shown. Scale bars, 200 μm. See also Figures S1, S2, and S4.

According to the correlation matrix, transcriptome changes associated with the overexpression of *Smad7*, *Nr2f1*, *Sox11*, *Dmrt1*, *Sox9*, *Foxg1*, *Klf3*, *Pou5f1*, *Sox2*, or *Ascl1* were all related to neural tissues/organs, such as spinal cord, cerebellum, and cerebral cortex (Figures 5A and S1) (Nishiyama et al., 2009; Correa-Cerro et al., 2011). As expected (Vierbuchen et al., 2010), overexpression of *Ascl1* produced TUJ1^+^ and MAP2^+^ neurons by day 5, which further increased by day 7 (Figure S4). FACS analysis showed that the overexpression of *Ascl1* significantly increased the number of PSA-NCAM^+^ neural progenitor cells, whereas it did not increase PDGFRα^+^ mesoderm cells and FOXA2^+^ endoderm cells (Figure S2C).

Use of neuron-specific medium further increased the efficiency of neural differentiation by *Ascl1* (Figure 5B). Neurons induced by *Ascl1* expressed a variety of neural markers: pan-neural markers (TUJ1, MAP2, and NEUN); a synaptic marker (SYNAPSIN); dopaminergic neuron markers (tyrosine hydroxylase [TH] and dopamine transporter [DAT]); a motor neuron marker (ISL1/ISL2); and an inhibitory neurotransmitter (GABA) (percentage of TH^+^ out of TUJ1+ population [TH^+^/TUJ1+] was 8.3% ± 1.0%, ISL1+/TUJ1+ was 37.6% ± 9.0%, and GABA+/TUJ1+ was 27.2% ± 8.9%, from three independent experiments) (Figures 5C and 5H).

To investigate the active and passive membrane properties of these neurons, we performed the patch-clamp recording of *Ascl1*-induced neurons on day 11: 5 out of the 11 examined cells fired action potentials that reached 0 mV upon injection of depolarizing currents (Figure 5D). Furthermore, the recorded cells showed some forms of synaptic activity that could be seen in both current clamp and voltage-clamp conditions (Figure 5E). Reversal potential values of synaptic activity were −19.9 ± 5.5mV and −38.6 ± 5.6 mV for cells that fire action potential and nonfiring cells (n = 5 and n = 4 [independent cell cultures]; p = 0.0393), respectively (Figure 5E). To block glutaminergic synaptic transmission in the cell firing action potentials, a selective NMDA receptor antagonist, 50 μM APV, and a selective AMPA receptor antagonist, 20 μM NBQX, were applied for 3–5 min. This caused a 17.3 ± 5.9 mV shift in the negative direction (three independent experiments), which is consistent with the fact that glutaminergic synapses are active in neurons differentiated from ESCs.

We also tested other TFs predicted by the correlation matrix: *Smad7* (Ozair et al., 2013), *Sox11* (Wang et al., 2013), *Sox9* (Scott et al., 2010), *Sox2* (Graham et al., 2003; Bani-Yaghoub et al., 2006), and *Foxg1* (Pratt et al., 2002; Miyoshi and Fishell, 2012), which have already been shown as TFs involved in the neurogenesis during development. FACS analysis showed that overexpression of any one of these TFs alone significantly induced PSA-NCAM^+^ neural progenitors, which correlate with neural differentiation (Figures 5F and 5G). Four additional TFs (*Nr2f1*, *Dmrt1*, *Klf3*, and *Pou5f1*) that the correlation matrix indicated as potential neural differentiation factors also turned out to be interesting examples. *Nr2f1* (also known as *Coup-TFI*) has been implicated for specifying neural stem cells (NSCs) (Naka et al., 2008; Lodato et al., 2011) and the migration of neurons (Zhou et al., 1999; Alfano et al., 2011). However, the roles of *Nr2f1* during early neurogenesis are not fully elucidated. Here, we found that *Nr2f1* overexpression significantly induced PSA-NCAM^+^ neural progenitors at the level comparable to the best inducers thus far: *Ascl1* and *Smad7* (Figures 5F and 5G). Interestingly, overexpression of *Nr2f1* significantly increased GABA^+^ inhibitory interneurons compared with neurons induced by *Smad7* (GABA+/Tuj1+; *Nr2f1* was 63.9% ± 13.4% versus *Smad7*, which was 8.7% ± 4.8%, from three independent experiments; ^∗^p < 0.05) (Figures 5H and 5I). Thus, we established that *Nr2f1* is a potent inducer of not only neural precursor but also inhibitory interneurons.

### Global Gene Expression Profiling and Direct Binding of TFs in Target Genes

**Figure 6:**
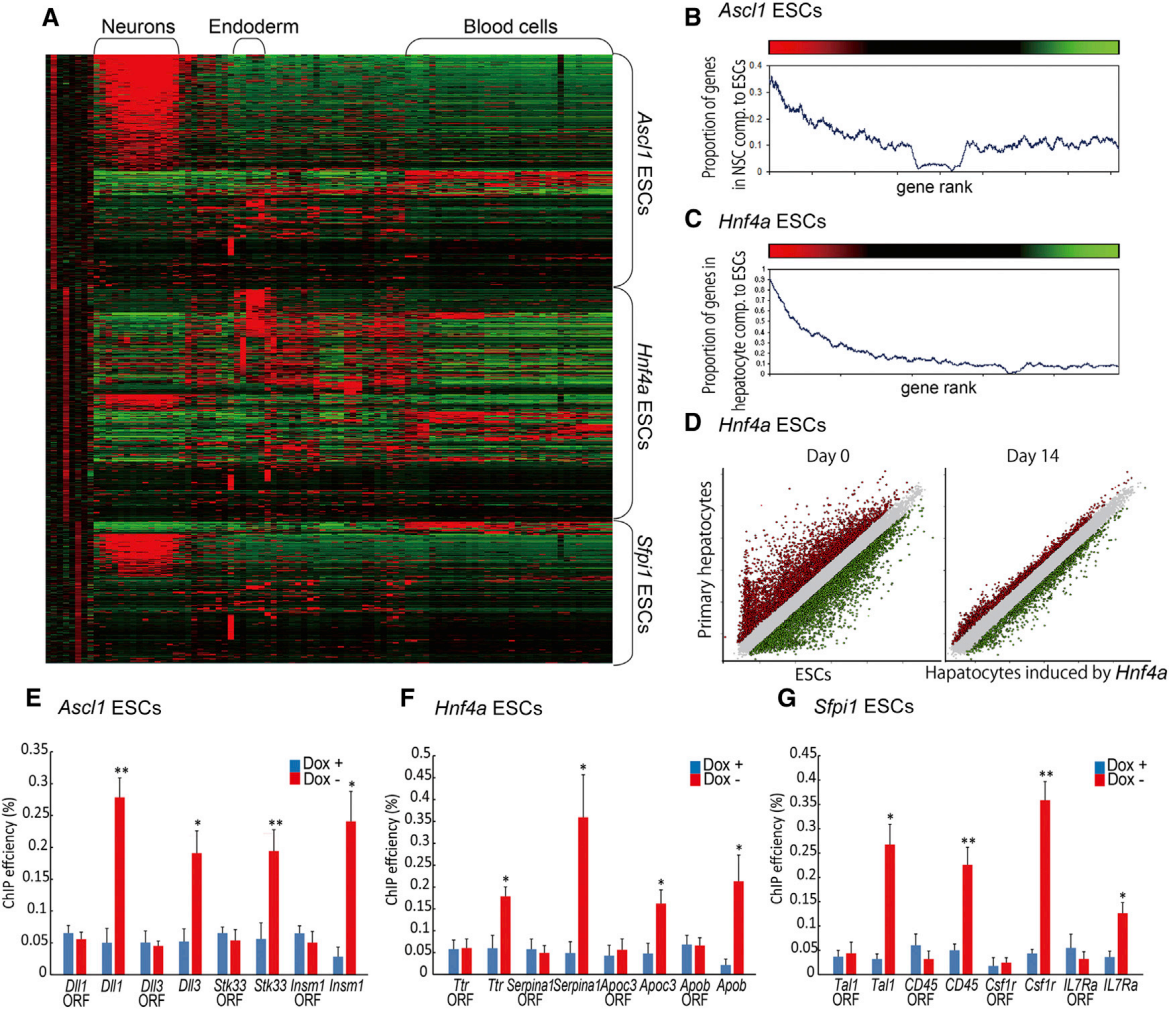
Characterization of TF-Induced Differentiated Cells by Global Gene Expression Profiling and ChIP (A) A heatmap shows the association of genes upregulated >2-fold after the induction of TFs—*Ascl1*, *Hnf4a*, and *Sfpi1* (upper, middle, and lower portions of the heatmap, respectively)—with transcriptomes of various tissues/organs in the GNF database. Red and green colors represent higher and lower gene expression levels, respectively. The first six columns show the changes of gene expression (log ratio between cells cultured in the Dox− and Dox+ conditions) after the induction of the TFs at day 3 (columns 1, 3, and 5) and day 7 (columns 2, 4, and 6). Other columns show the gene expression levels from the GNF database normalized to the median expression in all tissues/organs shown here. Hierarchical clustering was used to order both genes and tissue/organs from the GNF database and then the order was further edited manually. (B) Rank plot analysis compared the gene expression profile of neurons induced by *Ascl1* with that of NSCs derived from adult brains. Genes were sorted by expression changes after the induction of *Ascl1* on day 7 (from high expression, red, to low expression, green). Then, the proportion of genes that were upregulated >3-fold in the NSCs compared to ESCs was estimated in a sliding window of 500 genes. Statistical significance was z = 57.37 and p < 10^−100^ (PAGE test, Kim and Volsky 2005). (C) Rank plot analysis compared the gene expression profile of hepatocytes induced by *Hnf4a* with that of the primary hepatocyte culture. Genes were sorted by expression changes after the induction of *Hnf4a* on day 14. Then the proportion of genes that were upregulated >2-fold in hepatocytes compared to ESCs was estimated in a sliding window of 500 genes. Statistical significance was z = 138.96 and p < 10^−100^ (PAGE test). (D) Scatterplots compared gene expression profiles of primary hepatocytes with that of ESCs or hepatocytes induced by *Hnf4a* on day 14 of differentiation. (E–G) ChIP assays on neuron-related genes (E), hepatocyte-related genes (F), and blood cell-related genes (G) of day 7 of differentiation using ESC lines carrying *Ascl1* (E), *Hnf4a* (F), or *Sfpi1* (G) gene are presented. ORF primers were used as negative control (three independent experiments, SEM; ^∗^p < 0.05 and ^∗∗^p < 0.01 versus Dox+). See also Figures S5, S6, and Table S1.

To further examine the cell differentiation by the overexpression of these single TFs, we carried out the global gene expression profiling of ESC-derived differentiated cells. The transcriptome of ESCs shifted toward a neural fate after the induction of *Ascl1*, toward an endoderm fate after induction of *Hnf4a* and *Foxa1*, and toward a blood cell fate after induction of *Sfpi1* (Figures 6A and S5). Maturity of differentiated cells by TFs was assessed by gene rank plot analysis, which examined the association between a list of genes upregulated after the induction TFs and a list of genes specific to NSCs (Aiba et al., 2006) or primary hepatocytes. Rank plot analysis revealed that gene expression profile of *Ascl1*-induced neurons was similar to that of NSCs derived from adult mouse brains (Figure 6B). Furthermore, the gene expression profile of hepatocytes induced by *Hnf4a* from ESCs showed extensive similarity to that of the primary hepatocytes (Figures 6C and 6D). We further examined if *Ascl1*, *Hnf4a*, or *Sfpi1* directly binds to the promoter region of genes related to blood cells, hepatocytes, or neurons, as reported by Sladek et al. (1990), Metzger et al. (1993), Reddy et al. (1994), Bockamp et al. (1998), Anderson et al. (2001), DeKoter et al. (2002), and Castro et al. (2006), with the chromatin immunoprecipitation (ChIP) assay using FLAG antibody. We observed that the FLAG-tagged exogenous ASCL1 protein bound to the ASCL1-binding motif in the promoter regions of neuron-related genes (*Dll1*, *Dll3*, *Stk33*, and *Insm1*), the FLAG-tagged exogenous HNF4A protein bound to the HNF4A-binding motif in the promoter regions of hepatocyte-related genes (*Ttr*, *Serpina1*, *Apoc3*, and *Apob*), and the FLAG-tagged exogenous SFPI1 protein bound to the SFPI1-binding motif in the promoter regions of hematopoietic-related genes (*Tal1*, *CD45*, *Csf1r*, and *IL7Ra*) (Figures 6E–6G and S6). Taken together, the overexpression of single TFs orchestrates global gene expression changes through direct binding to the specific target genes.

### Effectiveness of Synthetic mRNAs for ESC Differentiation

**Figure 7:**
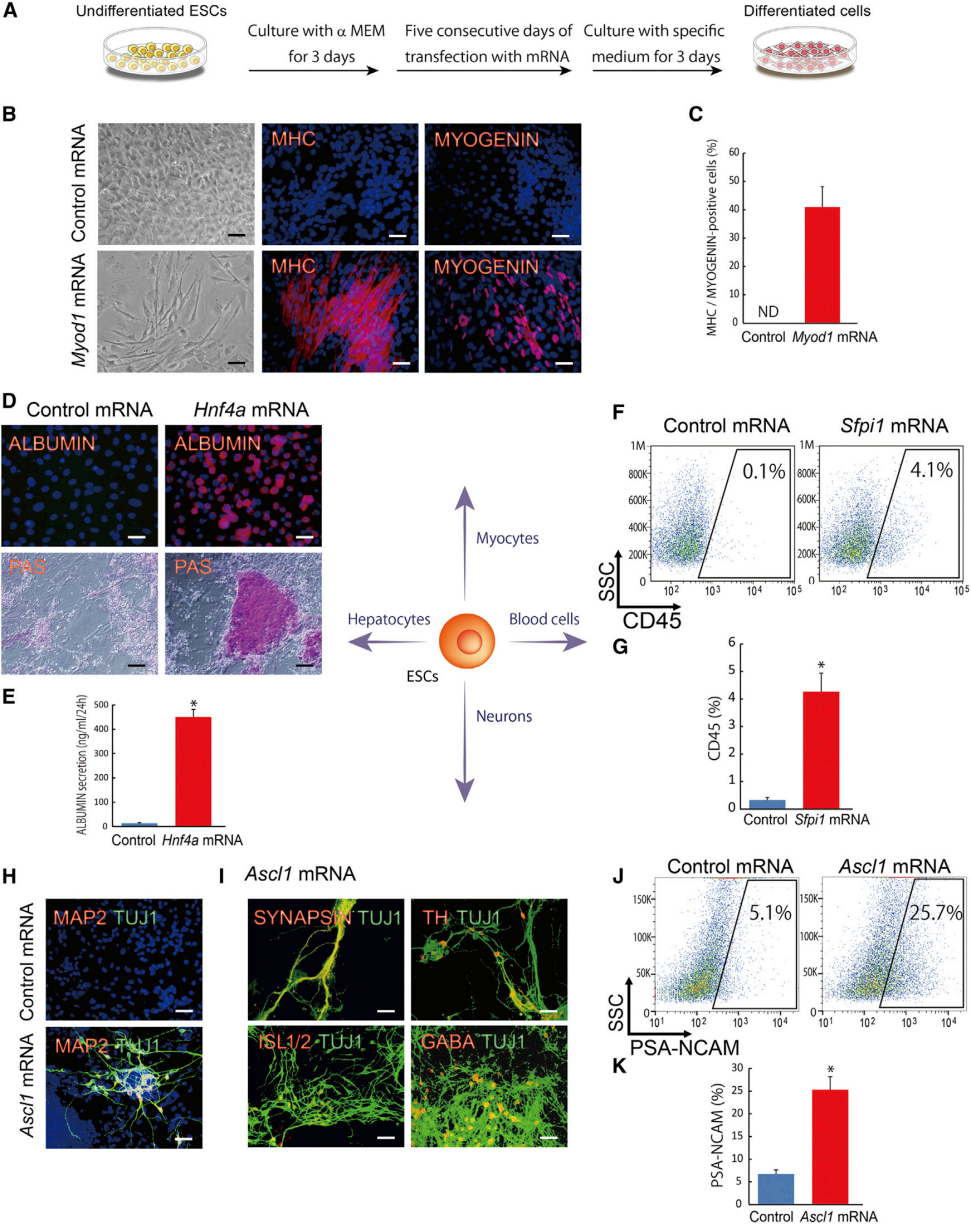
Synthetic mRNAs Induce Myocytes, Hepatocytes, Blood Cells, or Neurons from ESCs (A) A schematic representation shows the experimental design for synthetic mRNA transfection to generate target organ cells from ESCs. (B) Immunostaining for MHC and MYOGENIN at differentiation 11 days with *Myod1* mRNA is presented. Scale bars, 200 μm. (C) Quantitative evaluation of MHC^+^/MYOGENIN^+^ cell percentages on day 11 of differentiation with *Myod1* mRNA (three independent experiments, SEM) is shown. (D) Immunostaining for ALBUMIN and PAS staining on day 11 of differentiation with *Hnf4a* mRNA is presented. Scale bars, 200 μm. (E) Amounts of ALBUMIN in the culture media, measured on day 14 of *Hnf4a* mRNA-transfecting ESC differentiation (three independent experiments, SEM; ^∗^p < 0.01 versus control), are shown. (F and G) FACS analysis for CD45+ cell appearance with *Sfpi1* mRNA (three independent experiments, SEM; ^∗^p < 0.01 versus control) is presented. (H and I) Immunostaining for TUJ1, MAP2, SYNAPSIN, TH, ISL1/ISL2, or GABA at differentiation 11 days used *Ascl1* mRNA. Scale bars, 200 μm. (J and K) FACS analysis on day 8 of differentiation for PSA-NCAM^+^ cell appearance with *Ascl1* mRNA (three independent experiments, SEM; ^∗^p < 0.01 versus control) is shown. See also Figure S7.

An interesting feature of our strategy is the rapid differentiation of ESCs, requiring only several days of TF overexpression. We reasoned that this feature provides the optimum use of the synthetic mRNA transfection protocol because this DNA integration-free procedure is more suitable to potential therapeutic applications (Warren et al., 2010). To test this notion, ESCs were first cultured for 3 days in the DM (without LIF) and then subjected to 5 consecutive days of transfection with *Myod1*, *Hnf4a*, *Sfpi1*, or *Ascl1*-encoding RNA, followed by 3 days of cell culture in the DMs (Figure 7A). The transfection of synthetic mRNAs of defined TFs differentiated ESCs into the expected cell lineages: *Myod1* mRNA produced MHC^+^ and MYOGENIN^+^ myocytes in a dose-dependent manner (Figures 7B, 7C, and S7A); *Hnf4a* mRNA produced ALBUMIN- and PAS^+^ hepatocytes (Figures 7D and 7E); *Sfpi1* mRNA produced CD45^+^ hematopoietic cells (Figures 7F and 7G); and *Ascl1* mRNA produced TUJ1^+^, MAP2^+^, TH^+^, ISL1/ISL2^+^, or GABA^+^ neurons and also increased the number of PSA-NCAM^+^ neural progenitors (Figures 7H–K and S7B). The efficiencies of cell differentiation by the transfection a TF mRNA were lower by one-third to two-third than those obtained by the induction of TFs with tet-off system (Figures 7C, 7E, 7G, and 7K). The reduced efficiencies were most likely caused by the <100% transfection efficiency but also caused by the stress of mRNA transfection because even the transfection of control GFP mRNA reduced the efficiencies of lineage-specific cell differentiation (data not shown). Nevertheless, our results suggest that the transfection of TF mRNA can be used to induce lineage-specific cell differentiation.

## Discussion

Here, we have demonstrated the feasibility of systematic discovery of differentiation-directing single TFs based on the correlation matrix of global gene expression profiles. This approach provides a means to find TFs suitable to tip the balance of ESCs rapidly and effectively to discrete cell lineages. In addition to the demonstration of a proof of concept for such a strategy, our work here has revealed some interesting features of the TF networks that regulate ESC differentiation.

First, considering a prevailing methodology of using a combination of TFs to change the cell’s identity (Takahashi and Yamanaka, 2006; Feng et al., 2008; Vierbuchen et al., 2010; Caiazzo et al., 2011; Son et al., 2011; Sekiya and Suzuki, 2011; Huang et al., 2011), it is striking to find that the overexpression of a single TF not only initiates but also guides through the mature differentiation of ESCs with the help of additional environmental factors (e.g., lineage-specific cell differentiation culture media). Furthermore, the correlation matrix has identified TFs for cell differentiation: *Esx1* for myocyte differentiation, *Gata2* and *Gata3* for hepatocyte differentiation, *Elf5* for blood cell differentiation, and *Dmrt1* for neuron differentiation. Once a set of TFs, each of which can differentiate ESCs into the same cell/tissue type, is identified (Feng et al., 2008; Vierbuchen et al., 2010; Caiazzo et al., 2011; Son et al., 2011; Sekiya and Suzuki, 2011; Huang et al., 2011), it would be straightforward to test the combination of such TFs for possibly more effective ESC differentiation. More importantly, our method makes it possible to identify single TFs that can even specify subtypes of cells, as exemplified by the preferential generation of GABA^+^ inhibitory neurons by the overexpression of *Nr2f1*. This may eventually provide tools to fine-tune ESC differentiation to more specific cell types, e.g., inhibitory interneurons, pacemaker cells in heart, and B cells, which are required for cell therapies with ESCs and iPSCs.

Second, it is important to point out that in all cell lineages examined in this work, we have observed that a TF, which normally functions at the late stage of cell differentiation, initiates and guides through the differentiation of ESCs. Interestingly, our results suggest that if a powerful downstream TF is directly expressed, it can rapidly bypass (or alternatively, force any necessary backup for) TF activation cascades for cell differentiation and impel the further downstream events to complete the differentiation process. This could explain why the cell differentiation by the overexpression of a TF is faster than the cell differentiation by the manipulation of environmental conditions. Of course, direct reprogramming of fibroblast cells to specific cell types can be considered, in a sense, examples of bypassing the cell differentiation cascades; however, most cases require the combinations of multiple TFs (Takahashi and Yamanaka, 2006; Feng et al., 2008; Caiazzo et al., 2011; Son et al., 2011; Sekiya and Suzuki, 2011; Huang et al., 2011) except for *Myod1* (Davis et al., 1987) and *Ascl1* (Vierbuchen et al., 2010). Taken together, our results further support the notion that even single TFs can overwrite the wiring of genes in pluripotent stem cells.

Overall, our approach has the practical outcome of facilitating the finding of more rational routes to obtain desired differentiated cell types from pluripotent stem cells, such as ESCs and iPSCs. Such knowledge could lead to a novel strategy for possible future therapeutic applications.

## Experimental Procedures

### Cell Culture and Differentiation

ESC lines carrying a tetracycline-regulatable TF (Nishiyama et al., 2009; Correa-Cerro et al., 2011) were cultured and differentiated as described previously (Yamamizu et al., 2009, 2012a). The following organ-specific cell culture media were also used: StemPro-34 Serum Free Media (Invitrogen) for blood cells, Hepatocyte Culture Media Kit (BD Biosciences) for hepatocytes, and NeuroCult Differentiation Kit (STEMCELL Technologies) for neurons. In some experiments, the following factors were used: stem cell factor (100 ng/ml), IL-3 (1 ng/ml), IL-6 (5 ng/ml), IL-11 (5 ng/ml), G-CSF (20 ng/ml), M-CSF (10 ng/ml), Flt3 ligand (10 ng/ml), ERYTHROPOIETIN (4 U/ml), and THROMBOPOIETIN (5 ng/ml) (R&D Systems) for generation of blood cells; and HGF (20 ng/ml) and OSM (20 ng/ml) (R&D Systems) for generation of hepatocytes. Differentiated cells were examined by immunostaining and flow cytometric analysis.

### FACS Analysis

Cultured cells were harvested in DM or DM and culture medium for blood cells at 3, 4, 5, and 11 days after differentiation and stained with APC-conjugated CD45 antibody MoAb (eBioscience) and then subjected to analysis using Accuri C6 or FACSCanto II (Becton Dickinson) (Yamamizu et al., 2012b). For intracellular proteins, cultured cells by induction of *Foxa1*, *Hnf4a*, *Gata2*, *Gata3*, or *Gbx2* were fixed with 4% paraformaldehyde and washed by PBS with 5% FCS and 0.75% Saponin (Sigma-Aldrich) (Yamamizu et al., 2012a). Fixed cells were stained with PE-conjugated anti-FOXA2 antibody (Bioss) and then subjected to analysis using Accuri C6 or FACSCanto II.

### Immunohistochemistry

Immunostaining for cultured cells was carried out as described previously (Yamamizu et al., 2009, 2012a). Primary antibodies were as follows: mouse anti-MHC (1:500; DSHB); mouse anti-MYOGENIN (1:500; BD Biosciences); goat anti-SOX17 (1:500; R&D Systems); mouse anti-AFP (1:200; R&D Systems); mouse anti-ALBUMIN (1:200; R&D Systems); mouse anti-βIII-TUBULIN (TUJ1) (1:500; Covance); rabbit anti-TUJ1 (1:500; Covance); mouse anti-MAP2 (1:500; Sigma-Aldrich); mouse anti-NEUN (1:100; Millipore); mouse anti-SYNAPSIN I (1:100; Millipore); rabbit anti-TH (1:500; Millipore); rat anti-DAT (1:500; Millipore); mouse anti-iSL1/ISL2 (1:500; DSHB); and rabbit anti-GABA (1:500; Sigma-Aldrich).

### Coculture of Myocytes and C2C12 Cells

C2C12 cells were stained with a red PKH26 dye (Sigma-Aldrich) before coculturing with myocytes derived from ESCs. ESCs were differentiated into myocytes for 7 days by induction of *Myod1*-IRES-*Venus*. On day 7, medium was replaced with DMEM supplemented with 2% horse serum, and 1 × 10^5^ C2C12 cells were seeded onto myocytes. After 16 and 40 hr, C2C12 cell-stained PKH26 and myocyte-expressed *Myod1*-IRES-*Venus* were photographed with inverted fluorescent microscopy (Eclipse TE300; Nikon) with the use of NIS-Elements Software (Nikon).

### PAS Staining, LDL Uptake, and Albumin ELISAs

Cells on day 7 of differentiation were fixed by 4% paraformaldehyde and stained by PAS (Sigma-Aldrich) according to the manufacturer’s instructions. LDL uptake by cells was assessed by fluorescent microscopy after incubation of the differentiated cells with 10 μg/ml acetylated LDL labeled with 1,1′-dioctadecyl-3,3,3′,3′-tetramethylindo-carbocyanine perchlorate (DiI-Ac-LDL) (Biomedical Technologies) for 4 hr at 37°C and DAPI. The amounts of mouse ALBUMIN secreted in the culture media (DMEM without glucose or phenol red [Invitrogen] supplemented with 2 mM sodium pyruvate [Sigma-Aldrich] and 20 mM sodium lactate [Sigma-Aldrich]) were measured after culture of differentiated cells from ESCs or adult mouse primary hepatocytes for 24 hr using a Mouse Albumin ELISA Kit (Bethyl Laboratory) according to the manufacturer’s instructions. The absorbance was measured with a VICTOR^3^ V microplate reader (PerkinElmer).

### Hematopoietic Colony-Forming Cell Assay

Hematopoietic colony-forming cell assays were performed in MethoCult H3434 semisolid medium (STEMCELL Technologies) supplemented with 50 ng/ml human TPO and 10 ng/ml G-CSF. A total of 2 × 10^5^ differentiated cells in the presence (Dox+) or absence (Dox−) of doxycycline (1 μg/ml). ESCs were plated on day 3 of differentiation in 1.4 ml of hematopoietic colony-forming assay medium and cultivated for an additional 11 days (Yamamizu et al., 2012b). The colonies were then collected, stained with Hemacolor (Merck), and observed under a microscope.

### Electrophysiology

Whole-cell recordings were made from differentiated neurons plated on the culture dish (FluoroDish; World Precision Instruments) for 11 days. For visual guidance, we used an upright microscope equipped with DIC infrared camera as described previously by Belforte et al. (2010). The recording solution contained 130 mM NaCl, 3.5 mM KCl, 10 mM glucose, 1.25 mM NaH_2_PO_4_, 24 mM NaHCO_3_, 2 mM CaCl_2_, and 1 mM MgSO_4_ and was oxygenated continuously, and osmolality was set at 315 mOsm. The same internal solution was used in all the recordings, and it contained 125 mM KCl, 10 mM NaCl, 4 mM Mg-ATP, 0.3 mM Na-GTP, and 1.6 mM KHCO_3_ with a pH of 7.3, and osmolality was set at 270–290 mOsm. The recording solution was oxygenated continuously, and osmolality was set at 315 mOsm and kept at room temperature. Input resistance and membrane time constant were measured in current clamp conditions. Spontaneous events were collected semiautomatically during 30 s long periods at different holding potentials, and the ten largest ones observed at least 2× SD above the baselines were chosen to calculate the reversal potential values after fitting a linear line across points. Liquid junction potential was measured −5.5 mV (Neher, 1992) and subtracted offline to calculate reversal potential values of synaptic activity.

### Microarray Analysis

Microarray analyses were carried out as described previously by Nishiyama et al. (2009) and Correa-Cerro et al. (2011). Gene rank plots were used to check the association between gene expression change after the induction of Ascl1 and Hnf4a and genes specific for NSCs (Aiba et al., 2006) and primary hepatocytes. All genes were sorted by the decreasing expression change after the induction of TFs (i.e., log ratio of Dox− versus Dox+) and then the proportion of genes specific for NSCs or hepatocytes was estimated in a sliding window of 500 genes.

### ChIP

Differentiated cells after 7 days were subjected to crosslinking with 1% formaldehyde. Chromatin was digested in the buffer containing 0.1% sodium deoxycholate and then sheared to DNA fragments with an average length of 100–500 bp. Sonicated DNA was subjected to immunoprecipitation using anti-FLAG antibody (Sigma-Aldrich). Immunoprecipitated DNA was reverse crosslinked and then performed to qPCR using Power SYBR Green PCR Master Mix (Applied Biosystems). Sets of primers were used to amplify DNA sequences (Table S1). PCR amplification was conducted with a variable number of cycles (94°C for 30 s, 60°C for 30 s, and 72°C for 30 s).

### Synthesis of Modified mRNA and Transfection of mRNA

mRNA synthesis was performed as reported previously by Warren et al. (2010). ESCs were subjected to 5 consecutive days of transfection of *Myod1*, *Sfpi1*, *Hnf4a*, *Ascl1*-encoding RNA, or *GFP*-encoding RNA (control RNA) (Stemgent) with RNAiMAX from the third day of differentiation. After 5 consecutive days of transfection, cells were cultured in the organ-specific medium for 3 days. Differentiated cells were examined by immunostaining and flow cytometric analysis.

### Statistical Analysis

At least three independent experiments were performed. Statistical analysis of the data was performed with ANOVA. p < 0.05 was considered significant. Values are reported as mean ± SEM.

## Author Contributions

K.Y. performed all experiments and wrote the manuscript. Y.P. performed the microarray procedure. A.A.S. analyzed microarray data. V.Z. and K.N. carried out electrophysiology. Y.P. and H.Y. helped with mRNA synthesis. D.S. supervised the project. M.S.H.K. supervised all experiments and wrote the manuscript.

## Accession Numbers

The GEO accession number for microarray data reported in this paper is GSE43971.
