# Supplementary material for: Identification of Transcription Factors for Lineage-Specific ESC Differentiation
Source: Stem Cell Reports. 2013 Nov 27;1(6):545–59. doi: 10.1016/j.stemcr.2013.10.006 (PMC3871400; doi:10.1016/j.stemcr.2013.10.006)
Supplement: Document S1. Figures S1–S7, and Table S1 [file mmc1.pdf]

Stem Cell Reports, Volume *1*

Supplemental Information

Identification of Transcription Factors for Lineage-Specific ESC Differentiation

Kohei Yamamizu, Yulan Piao, Alexei A. Sharov, Veronika Zsiros, Hong Yu, Kazu Nakazawa, David Schlessinger, and Minoru S.H. Ko

Inventory of Supplemental Information

1. Supplementary Figure S1-S7

Supplemental Figure S1. Correlation of gene expression response to the induction of TFs with tissue-specific gene expression. – related to Figure 1-5

Supplemental Figure S2. FACS analysis for mesoderm, endoderm, and neural progenitor markers using ES cells carrying *Myod1, Hnf4a,* or *Ascl1* gene – related to Figure 2, 3, and 5.

Supplemental Figure S3. Immunostaining for endoderm and hepatocyte markers, at differentiation 7 days using ES cells carrying *Hnf4a, Foxa1, Gata2, Gata3,* or *Gbx2* gene – related to Figure 3.

Supplemental Figure S4. Immunostaining for pan-neuron markers at differentiation 5 and 7 days using ESCs carrying *Ascl1* gene – related to Figure 5.

Supplemental Figure S5. Heat map from microarray data demonstrating global gene expression pattern in differentiated cells by induction of *Foxa1* – related to Figure 6.

Supplemental Figure S6. qPCR for linage specific-genes at differentiation 7 days – related to Figure 6.

Supplemental Figure S7. Myogenic cells or neurons are induced by synthetic mRNA in a dose-dependent manner – related to Figure 7.

2. Supplemental Table

Supplemental Table 1. Primer list for ChIP – related to Figure 6.

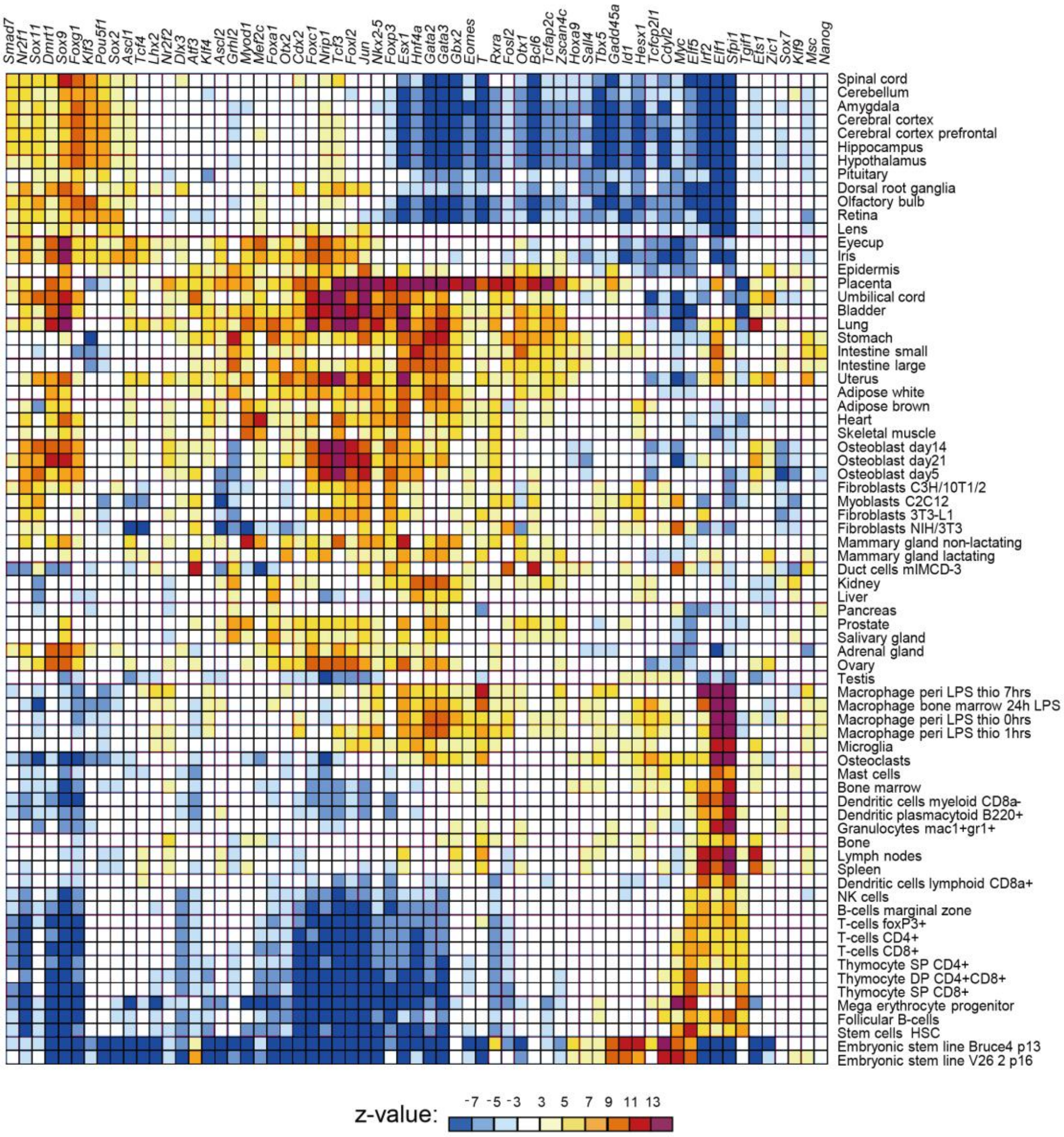

Supplemental Figure 1 Correlation of gene expression response to the induction of TFs with tissue-specific gene expression from the GNF ver.3 database (Figure reproduced from Correa-Cerro et al., 2011).

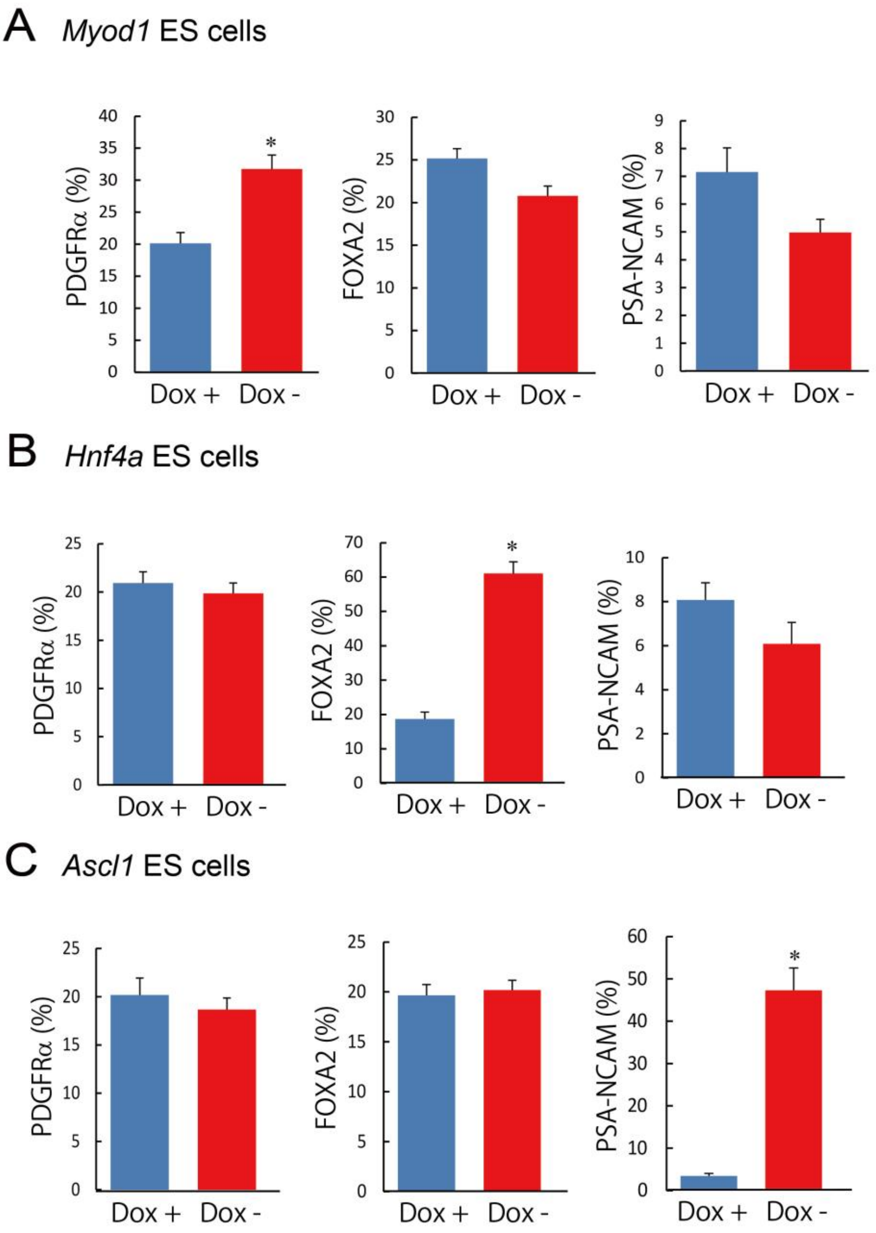

Supplemental Figure 2 FACS analysis for mesoderm, endoderm, and neural progenitor markers using ES cells carrying *Myod1*, *Hnf4a*, or *Ascl1* gene.

FACS analysis for PDGFRα (mesoderm marker), FOXA2 (endoderm marker) at differentiation 5 days, and PSA-NCAM (neural progenitor marker) at differentiation 6 days using ES cells carrying *Myod1* (A), *Hnf4a* (B), or *Ascl1* (C) gene (n=3 (independent experiments); *P<0.05 vs Dox+).

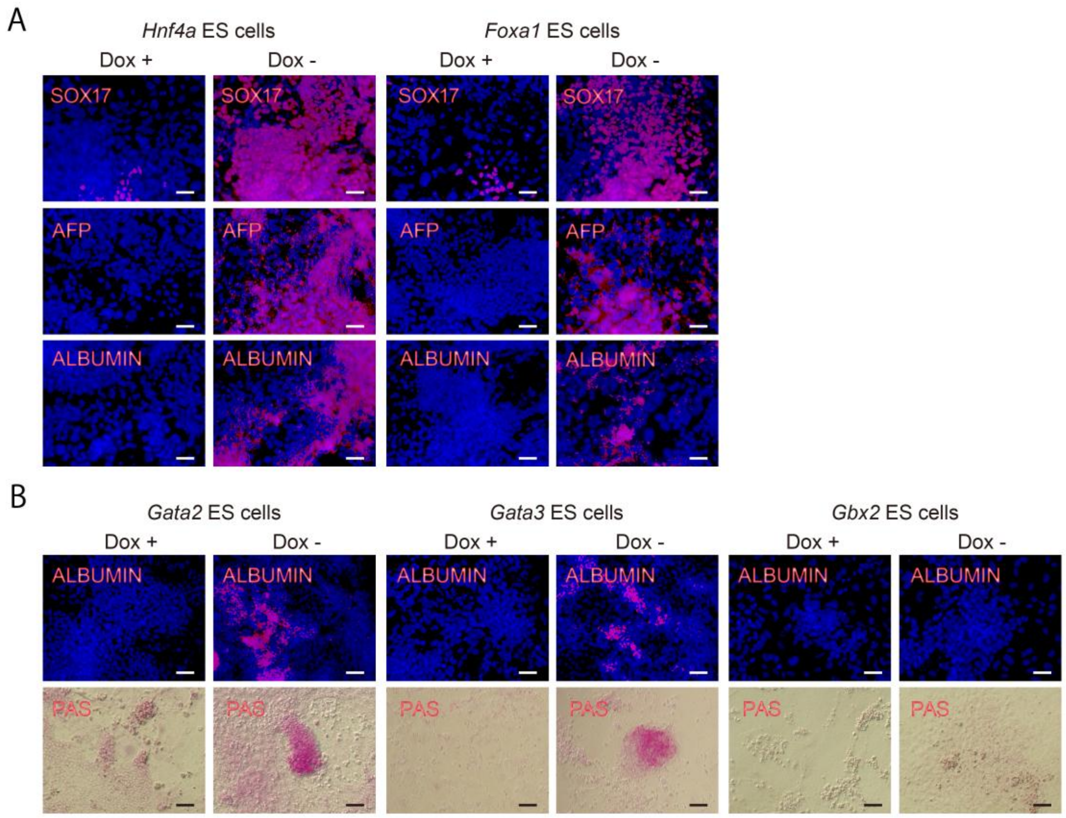

Supplemental Figure 3 Induction of *Hnf4a*, *Foxa1*, *Gata2*, or *Gata3* generates hepatocytes from ES cells.

(A) Immunostaining for SOX17 or α-fetoprotein (AFP), endoderm markers, and albumin, a hepatocyte marker, at differentiation 7 days using ES cells carrying *Hnf4a* or *Foxa1* gene. Scale bar: 200 μm. (B) Immunostaining for ALBUMIN, PAS staining, hepatocyte markers, at differentiation 7 days using ES cells carrying *Gata2*, *Gata3*, or *Gbx2* gene.

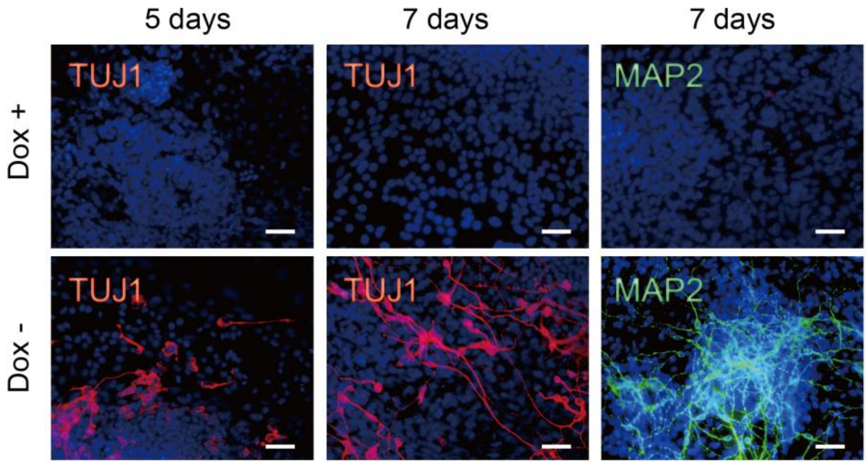

Supplemental Figure 4 Induction of *Ascl1* generates neurons from ES cells.
Immunostaining for βIII-tubulin (TUJ1) and MAP2, pan-neuron markers, with DM at differentiation 5 and 7 days using ESCs carrying *Ascl1* gene. Upper panels, Dox+. Lower panels, Dox-. Scale bar: 200 µm.

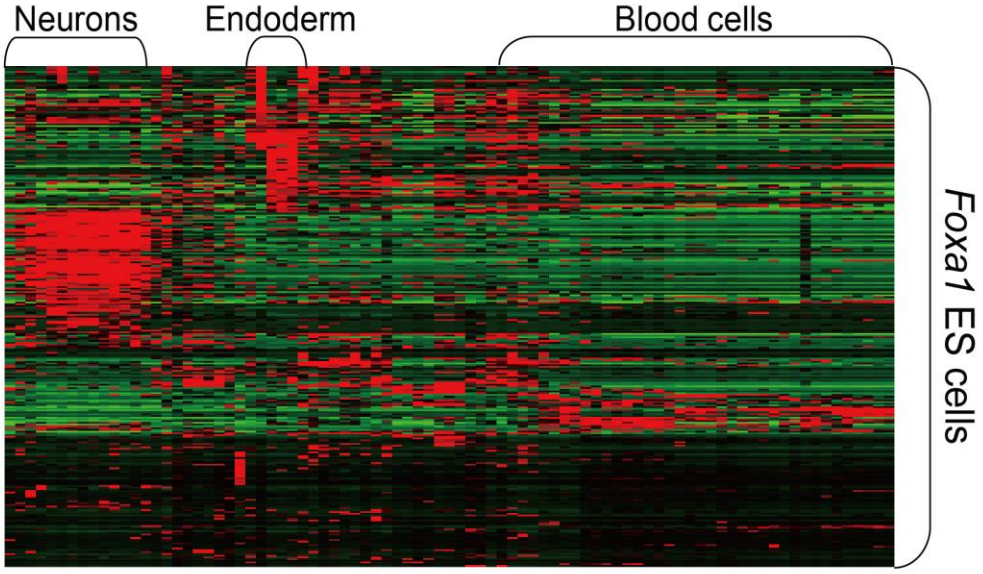

Supplemental Figure 5 ***Foxa1*** **associates with endoderm gene expression.**

Heat map from microarray data demonstrating global gene expression pattern in differentiated cells by induction of *Foxa1*. Red and green colors represent higher and lower gene expression levels, respectively.

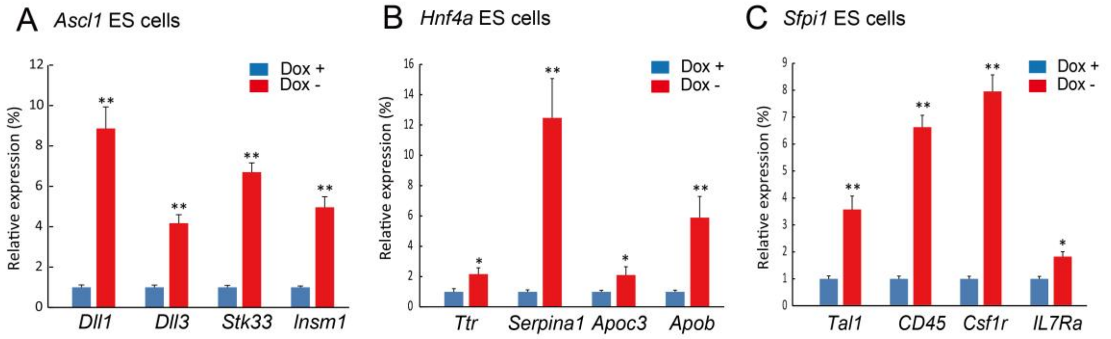

Supplemental Figure 6 Induction of *Ascl1, Hnf4a,* or *Sfpi1* increases organ-specific genes in differentiated cells.

(A) qPCR for neuron specific-genes at differentiation 7 days using ES cells carrying *Ascl1* gene (n=3 (independent experiments); **P<0.01 vs Dox+), (B) qPCR for hepatocyte specific-genes at differentiation 7 days using ES cells carrying *Hnf4a* gene (n=3; **P<0.01, *P<0.05 vs Dox+), (C) qPCR for blood cell specific-genes at differentiation 7 days using ES cells carrying *Sfpi1* gene (n=3 (independent experiments); **P<0.01, *P<0.05 vs Dox+).

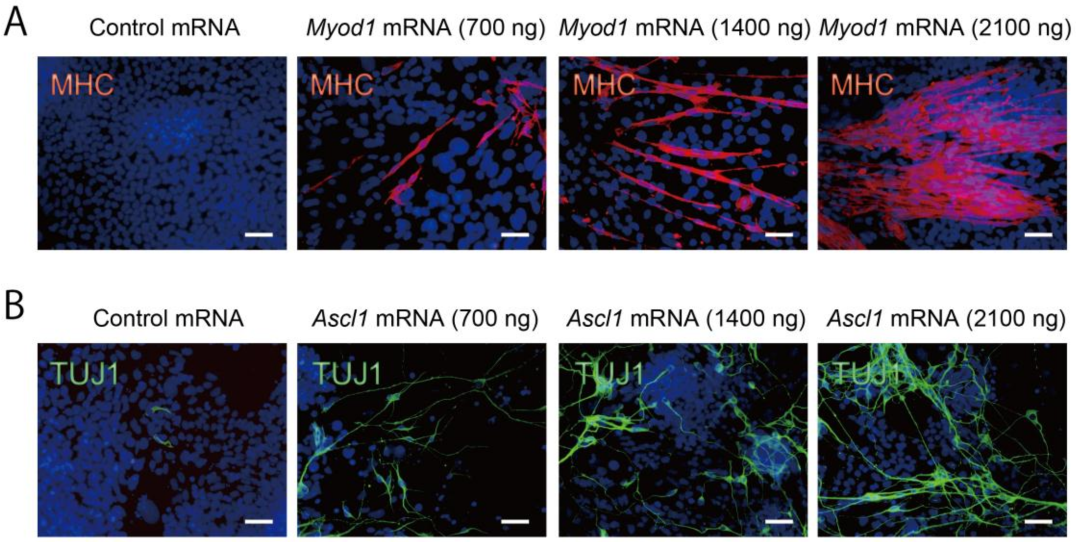

Supplemental Figure 7 Transfection of *Myod1* mRNA or *Ascl1* mRNA induces myogenic cells or neurons, respectively, from ESCs in a dose-dependent manner.

(A) Immunostaining for myosin heavy chain (MHC), a skeletal muscle marker, at differentiation 11 days with *Myod1* mRNA. Scale bar: 200 μm. (B) Immunostaining for βIII-tubulin (TUJ1), a pan-neuron marker, at differentiation 11 days with *Ascl1* mRNA. Scale bar: 200 μm.

ChIP Dll1 F	gcgtggctgtcattaagg
ChIP Dll1 R	ggtgctgtctgcattacc
ChIP Dll3 F	atttcctgtccgtttgcctctc
ChIP Dll3 R	gtaaatgtcgccatctgc
ChIP Stk33 F	acagctgctggagagaggac
ChIP Stk33 R	acttgtcccaagcctctgtg
ChIP Insm1 F	ttgggtgagcctgtcttag
ChIP Insm1 R	ccggccttatcttcacttc
ChIP Ttr F	aatctccctaggcaaggttca
ChIP Ttr R	tataccccctccttccaacc
ChIP Serpina1 F	gagcaaacagagaggggcta
ChIP Serpina1 R	agggatgggtgttctgactg
ChIP Apoc3 F	caggggcattacctggagta
ChIP Apoc3 R	ctcaggctctggtctggact
ChIP Apob F	tgagaccaccatcagatcca
ChIP Apob R	aggaggagctggcttaagga
ChIP Tal1 F	cggggagactctcttccttc
ChIP Tal1 R	ctcacgcaagcactctcaac
ChIP CD45 F	gggtcctctttgcaggaagt
ChIP CD45 R	agacgaaccgctaacagcat
ChIP Csf1r F	ttccctttcaggcaacctaa
ChIP Csf1r R	gctcccagctgctgctagttctg
ChIP IL7Ra F	gagagagggagacccaaacc
ChIP IL7Ra R	tctgcttacagcagcaatcc

Supplemental Table 1: Primer list for ChIP
